# Supplementary material for: Comparison of Broth Microdilution, Disk Diffusion and Strip Test Methods for Cefiderocol Antimicrobial Susceptibility Testing on KPC-Producing Klebsiella pneumoniae
Source: Antibiotics (Basel). 2023 Mar 20;12(3):614. doi: 10.3390/antibiotics12030614 (PMC10045316; doi:10.3390/antibiotics12030614)

Figure S1. CFD and CAZ-AVI MIC values distribution among CFD resistant KPC-Kp strains.

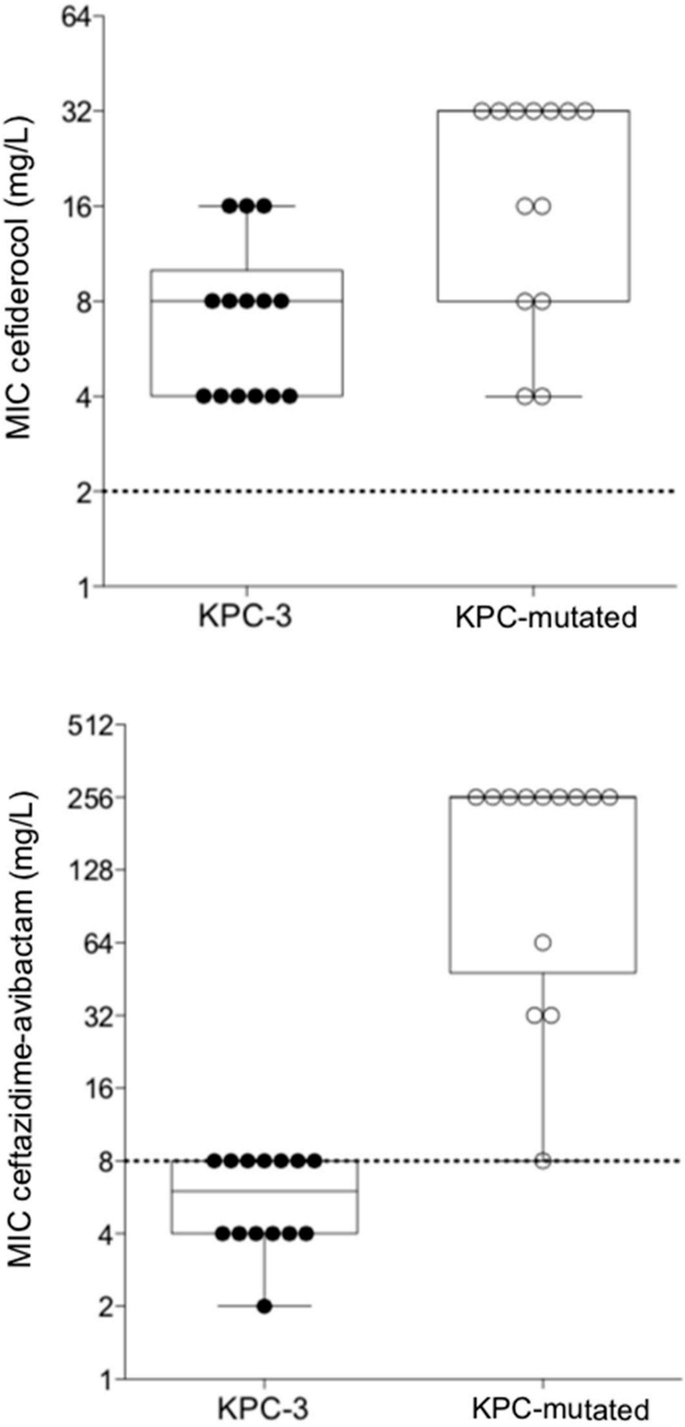

Supplement: Supplementary file 1 [file antibiotics-12-00614-s001.zip › antibiotics-2216183-supplementary.pdf]
